# Hormonal Responses to a Short Daytime Fast in Children With Beta‐Oxidation Disorders

**DOI:** 10.1002/jmd2.70120

**Published:** 2026-08-23

**Authors:** David Olsson, Maria Halldin, Charlotte Bieneck Haglind, Svetlana Lajic, Anna Nordenström

**Affiliations:** ^1^ Department of Women's and Children's Health, Unit for Pediatric Endocrinology Karolinska Institutet Stockholm Sweden; ^2^ Department of Pediatric Endocrinology and Inborn Errors of Metabolism, Astrid Lindgren Children's Hospital Karolinska University Hospital Stockholm Sweden; ^3^ Center for Inherited Metabolic Diseases, CMMS Karolinska University Hospital Stockholm Sweden

**Keywords:** beta‐oxidation disorders, fasting, fatty acid oxidation disorders, hormones

## Abstract

Fasting‐induced cellular energy deficiency and hypoglycemia are well‐known complications of beta‐oxidation disorders. The changes in energy homeostasis during fasting are under hormonal regulation. This aspect of fasting metabolism in beta‐oxidation disorders is not fully characterized. This clinical study investigates hormonal and substrate profiles during fasting and after a standardized meal in patients with beta‐oxidation disorders compared with age‐matched healthy controls. Children with beta‐oxidation disorders (4 LCHADD, 5 VLCADD, 6 MCADD, median age 8.5, 3–13 years) and healthy age‐matched controls (*n* = 12, median age 9.5, 5–13 years) were subjected to a 4‐h daytime fast. Blood samples for glucose, ketones, amino acids, nonesterified fatty acids (NEFA), acylcarnitines, and a panel of metabolically active hormones were analyzed after a 4‐h fast and 60 min after intake of the standardized meal. Patients with a beta‐oxidation disorder displayed a normal glycemic response to fasting. Most hormonal changes were similar to the changes observed in controls. However, children with LCHADD had higher insulin levels and BMI but, interestingly, relatively low HbA1c compared to children with VLCADD and MCADD. Our findings regarding a possible increased risk of insulin resistance in patients with LCHADD need to be verified in larger cohorts.

## Key Points

- Most hormonal changes in children with a beta‐oxidation disorder were like those observed in controls both during fasting and postprandially.
- A possible increased risk for insulin resistance in children with LCHADD should be further investigated.

## 1 Introduction

Mitochondrial fatty acid oxidation (FAO) is the main metabolic pathway for cellular energy homeostasis during fasting in humans [1]. More than 20 different enzymes and transport proteins are involved in this pathway, and pathogenic variants in the genes encoding the enzymes in the mitochondrial beta‐oxidation cycle result in monogenic recessive diseases belonging to the group of FAO disorders [2]. Several of the disorders are included in the neonatal screening program in many countries [3, 4].

Common to all beta‐oxidation disorders is deficient cellular energy production, but there are differences in symptoms related to the specific enzyme deficiencies that may be caused by toxic effects of different fatty acid intermediates. Organs with a high energy demand and/or a preference for generating energy from FAO, such as the CNS, skeletal muscles, liver, or heart, are especially vulnerable. In long‐chain 3‐hydroxyacyl‐CoA dehydrogenase deficiency (LCHADD), pathogenic variants in the *HADHA* gene result in an impaired ability to oxidize fatty acids of 16–18 carbon lengths [5]. Toxic effects of accumulating lipid metabolites in cardiac muscle have been described in both patients with LCHADD and in animal models [6, 7]. In addition, LCHADD is characterized by a progressive disease course, and the patients are at risk of developing retinopathy and peripheral neuropathy [8].

Very long‐chain acyl‐CoA dehydrogenase deficiency (VLCADD) leads to impaired oxidation of long‐chain fatty acids of 14–20 carbon lengths. The clinical spectrum in VLCAD deficiency ranges from severe neonatal onset to milder myopathic forms and asymptomatic phenotypes identified by newborn screening (NBS) [9]. Patients with medium‐chain acyl‐CoA dehydrogenase deficiency (MCADD) have a reduced ability to oxidize medium‐chain fatty acids. MCADD is characterized by fasting intolerance, and patients can develop hypoketotic hypoglycemia during intercurrent illness or prolonged fasting, which may be fatal if untreated [10]. Prolonged fasting is a risk factor for metabolic decompensation in beta‐oxidation disorders [11], and treatment guidelines include maximal fasting intervals and treatment options to avoid energy deficiency during intercurrent illness [12, 13]. For patients with symptomatic long‐chain beta‐oxidation disorders, dietary treatment with restriction of the intake of long‐chain triglycerides (LCT) and supplementation of medium‐chain triglycerides (MCT) is recommended, while patients with MCADD do not have restrictions in LCT intake or MCT supplementation [11, 14].

While many of the biochemical and clinical abnormalities that arise from beta‐oxidation disorders have been elucidated, the potential effect on the hormonal regulation of fasting is less clear. Epinephrine, cortisol, glucagon, and growth hormone (GH) are well‐known counter‐regulatory hormones preventing hypoglycemia and stimulating lipolysis. In a study of patients with LCHAD deficiency performed by our group, an increase in heart rate and a GH peak after 3 h of fast preceded an increase in subcutaneous glycerol and C:18 acylcarnitine species, indicating increased lipolysis [15]. In a study by Stenlid et al. [16], patients with VLCADD had lower levels of glucagon compared to patients with MCADD and carnitine uptake deficiency (CUD), suggesting that impaired glucagon secretion could play a role in the etiology of the propensity for hypoglycemia in this patient group.

A possible risk of developing insulin resistance in patients with beta‐oxidation disorders because of the dietary treatment with frequent meals and a relatively high intake of carbohydrates has been discussed. In addition, reduced FAO has been reported to increase insulin resistance [17]. While insulin resistance and development of Type 2 diabetes (T2D) remain potential long‐term risks for patients with beta‐oxidation disorders, a study in patients with LCHADD identified a normal glycemic response and insulin sensitivity in an oral glucose tolerance test [18].

Investigations of fasting metabolism in patients with beta‐oxidation disorders typically focus on nighttime fasting, as this is usually the longest fasting interval during the day [19, 20, 21]. The nighttime fasting intervals for individual patients with beta‐oxidation disorders will vary depending on the specific diagnosis, age, severity of the disease, and nutritional interventions such as the addition of corn starch to evening meals or gastrostomy feeds during the night.

Given that daytime fasting intervals in children with beta‐oxidation disorders are typically limited to 3–5 h depending on age and diagnosis, we examined the hormonal response to a 4‐h daytime fast following a standardized meal in children with LCHADD, VLCADD, and MCADD, as well as in healthy age‐matched controls.

## 2 Materials and Methods

### 2.1 Participants

Study participants were recruited from the pediatric metabolic units at the Karolinska University Hospital and the Skåne University Hospital in Sweden. Control subjects were recruited by local advertising at the Karolinska University Hospital. Exclusion criteria were signs of current infection or biochemical or clinical signs of metabolic decompensation in patients. One of the participants with LCHADD had a history of chronic alanine aminotransferase (ALT) elevation and had a value just above the normal range at the time of the study. All other participants had normal transaminase and creatinine kinase (CK) levels. In total, 27 participants were included in the study.

### 2.2 Study Protocol

Study participants were contacted before the day of the study and were prescribed a morning and a lunch meal based on their dietary preferences, weight, and age. The morning meal contained 10% of the recommended age‐adjusted daily energy intake (RDI), and the lunch meal contained 30% of the age‐adjusted RDI [22]. Patients with LCHADD and three patients with VLCADD had medium chain triglyceride (MCT) supplementation to one or both meals, constituting 7–24 energy percent of the meals, while all other participants had only LCT‐containing meals (see Supporting Information Appendix A1). Nutrient compositions of the study meals were calculated using the DietistNet software (Kost och Näringsdata, Bromma, Sweden). The meals were isocaloric and with age‐adjusted nutrients. The breakfast provided 22% (12–29) of total energy intake from fat (LCT + MCT), 62% (51–69) from carbohydrates, and 18% (7–29) from protein. Lunch meals contained 61% (51–68) carbohydrates, 20% (13–31) protein, and 20% (13–31) fat (LCT + MCT) (Supporting Information Appendix A1).

**FIGURE 1:**
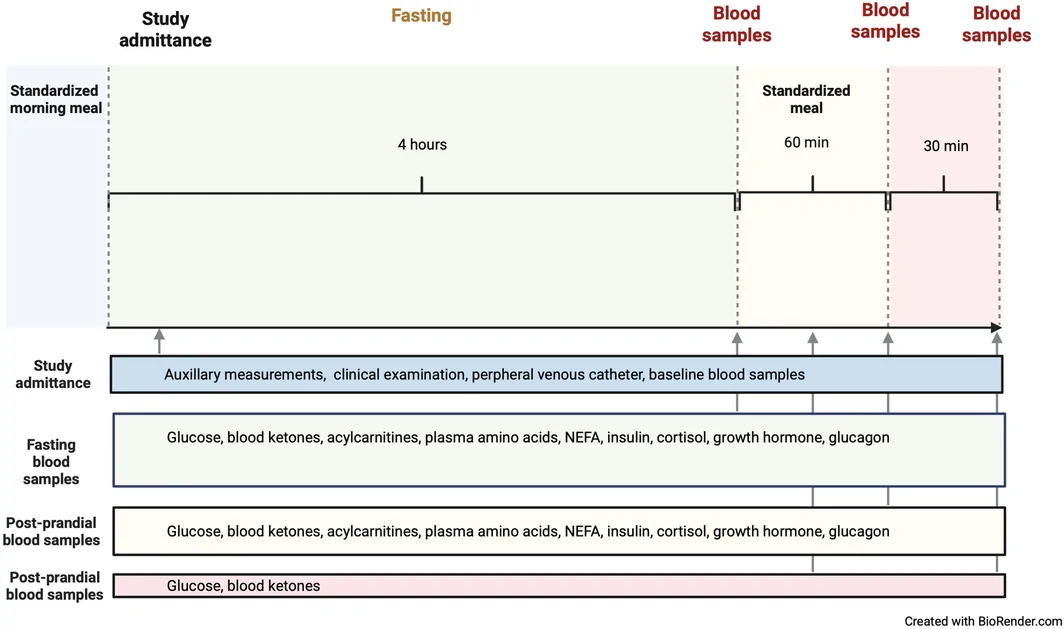
Study protocol.

After consuming their prescribed morning meal at home, study participants were admitted to the pediatric metabolic outpatient clinic. A venous catheter was placed after application of local anesthetic (EMLA, AstraZeneca, Cambridge, UK), and blood samples were taken for analysis of CK, ALT, aspartate aminotransferase (AST) (to exclude metabolic decompensation), and glycosylated hemoglobin (HbA1c). Blood samples were drawn 4 h postprandial, before lunch, and 60 min after the standardized lunch meal. The samples were analyzed for analyses of glucose, ketones, insulin, cortisol, GH, plasma acylcarnitines, plasma amino acids, plasma nonesterified fatty acids (NEFA), and plasma glucagon. In addition, capillary blood glucose and capillary blood ketones were analyzed 30, 60, and 90 min after the lunch meal was served (Figure 1).

### 2.3 Biochemical Analysis

Plasma acyl carnitines were analyzed using tandem mass spectrometry on Xevo TQ or Xevo TQ‐S from Waters, using a modified protocol by Goshal et al. [23]. Plasma amino acids were analyzed using ion exchange chromatography on Biochrom 30+ from Biochrom Ltd. using a modified protocol by Hommes [24]. Blood levels of CK, ALT, AST, c‐peptide, HbA1c, cortisol, and GH were analyzed at the Department of Clinical Chemistry, Karolinska University Hospital. Capillary blood glucose and capillary blood ketones were analyzed bedside (HemoCue Glucose 201, Hemocue AB, Ängelholm, Sweden, and Freestyle Optium Neo Meter, Abbott Laboratories, Abbott Park, IL, USA). Analysis of plasma NEFA was performed at the Center for Inherited Metabolic Diseases, Karolinska University Hospital, using an enzymatic assay from Fujifilm (NEFA‐HR(2); Fujifilm, Wako Chemicals Europe GmbH, Neuss, Germany) according to the manufacturer's instructions. Blood samples for analysis of glucagon were immediately placed on ice and centrifuged at 2500 × *g* for 10 min, and plasma was stored at −80°C. Glucagon was measured with a commercially available ELISA kit (ref. 10‐1271‐01) from Mercodia AB, Uppsala, Sweden.

### 2.4 Statistical Analysis

Statistical analysis was performed using Prism 9 (GraphPad Software LLC, San Diego, CA, USA) and IBM SPSS Statistics version 26 (IBM Corporation, Armonk, NY, USA). Nonparametric statistics were used: The Spearman's rank correlation coefficient, the Kruskal–Wallis test between groups, the Mann–Whitney *U* test between two independent variables, and the Wilcoxon signed‐rank test to test differences between matched variables. All statistical tests were two‐sided, and a *p* ≤ 0.05 was considered significant. Results are presented as median and range. The data presented is analyzed for the three diagnostic groups and controls.

## 3 Results

### 3.1 Study Cohort

Four patients had LCHADD (median age 9.3 years [3–12.5]), including one patient with multiple trifunctional protein deficiency (MTPD); 5 patients had VLCADD (median age 9.5 years [6–11]); 6 patients had MCADD (median age 6.5 years [4–13.5]); and 12 healthy children made up the control group (median age 9.5 years [5.5–13]) (Table 1). The median BMI‐SDS for the LCHADD group was 1.9 (0.1–2.7), for the VLCADD group 1.1 (0.8–2.8), for the MCADD group −0.3 (−1.2 to 0.9), and for the controls 0 (−0.9 to 2). One patient with LCHADD, one with MCADD, and one of the controls had entered puberty. All patients had been genotyped as per clinical diagnostic routine, and for patients with VLCADD, the residual VLCAD enzyme activity was determined in lymphocytes as described previously [25] with minor modifications [26] (Table 1).

**TABLE 1:** Study cohort characteristics.

Patient	Diagnosis	Age at study (years)	BMI‐SDS at study	Mutation allele 1	Mutation allele 2	Residual enzyme activity (%)^a^	Clinical description
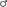	LCHADD	3	0.1	c.1209delA	c.1528G>C		Gastrostomy, night feeds, several rhabdomyolysis episodes^b^
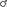	LCHADD	10.5	2.6	c.1528G>C	c.1528G>C		Gastrostomy, night feeds, several rhabdomyolysis episodes^b^
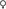	LCHADD	8	2.7	c.1528G>C	c.1528G>C		Gastrostomy, night feeds, several rhabdomyolysis episodes^b^
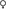	MTPD	12.5	1.1	c.703C>T^c^	c.703C>T^c^		Peripheral neuropathy, gastrostomy and night feeds, several rhabdomyolysis episodes^b^
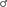	VLCADD	9.5	1.1	c.848T>C	c.1816T>C	4.5	Two rhabdomyolysis episodes^b^
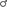	VLCADD	11	0.8	c.848T>C	c.1837C>T	6.6	Three rhabdomyolysis episodes^b^
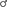	VLCADD	7	2.8	c.1678+IG/C	c.1591C>T	36.9	Mild phenotype, no rhabdomyolysis episodes^d^
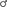	VLCADD	11	0.8	c.848T>C	c.1838G>A	12.5	No rhabdomyolysis episodes^d^
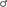	VLCADD	6	2.1	c.848T>C	c.388‐3T>G	7.4	Severe phenotype, neonatal hypoglycemia, frequent rhabdomyolysis episodes^b^
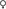	MCADD	8.5	−0.3	c.985A>G	c.985A>G		Mild clinic, carnitine supplementation^d^
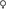	MCADD	4	−0.2	c.985A>G	c.985A>G		Mild clinic, carnitine supplementation^d^
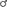	MCADD	4.5	−0.9	c.985A>G	c.199T>C		Asymptomatic^d^
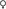	MCADD	4.5	−1.2	c.985A>G	c.199T>C		Asymptomatic^d^
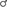	MCADD	10.5	0.9	c.985A>G	c.199T>C		Mild clinic, carnitine supplementation^d^
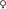	MCADD	13.5	0.7	c.985A>G	c.199T>C		Mild clinic, carnitine supplementation^d^
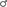	Control	7.5	−0.3				
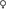	Control	5.5	0.1				
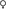	Control	10.5	1				
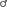	Control	8	0.6				
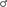	Control	10.5	−0.8				
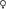	Control	10.5	−0.1				
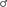	Control	11.5	−0.9				
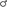	Control	13	0.1				
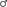	Control	10	2				
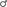	Control	9	−0.6				
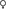	Control	7.5	−0.3				
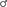	Control	7	0.4				

^a^ Palmitoyl‐CoA oxidation rate, percentage of healthy controls.

^b^ Strict diet (defined as LCT‐intake restricted to < 20% of total daily energy intake).

^c^ HADHA variant.

^d^ Relaxed diet.

All patients with LCHADD and three patients with VLCADD had dietary treatment with 10%–20% of total caloric intake from LCT fat and were prescribed MCT‐oil supplements. The patients with MCADD had no dietary restrictions but were prescribed extra carbohydrates and limited fasting periods during infections (Table 1).

### 3.2 Substrate Analyses

**FIGURE 2:**
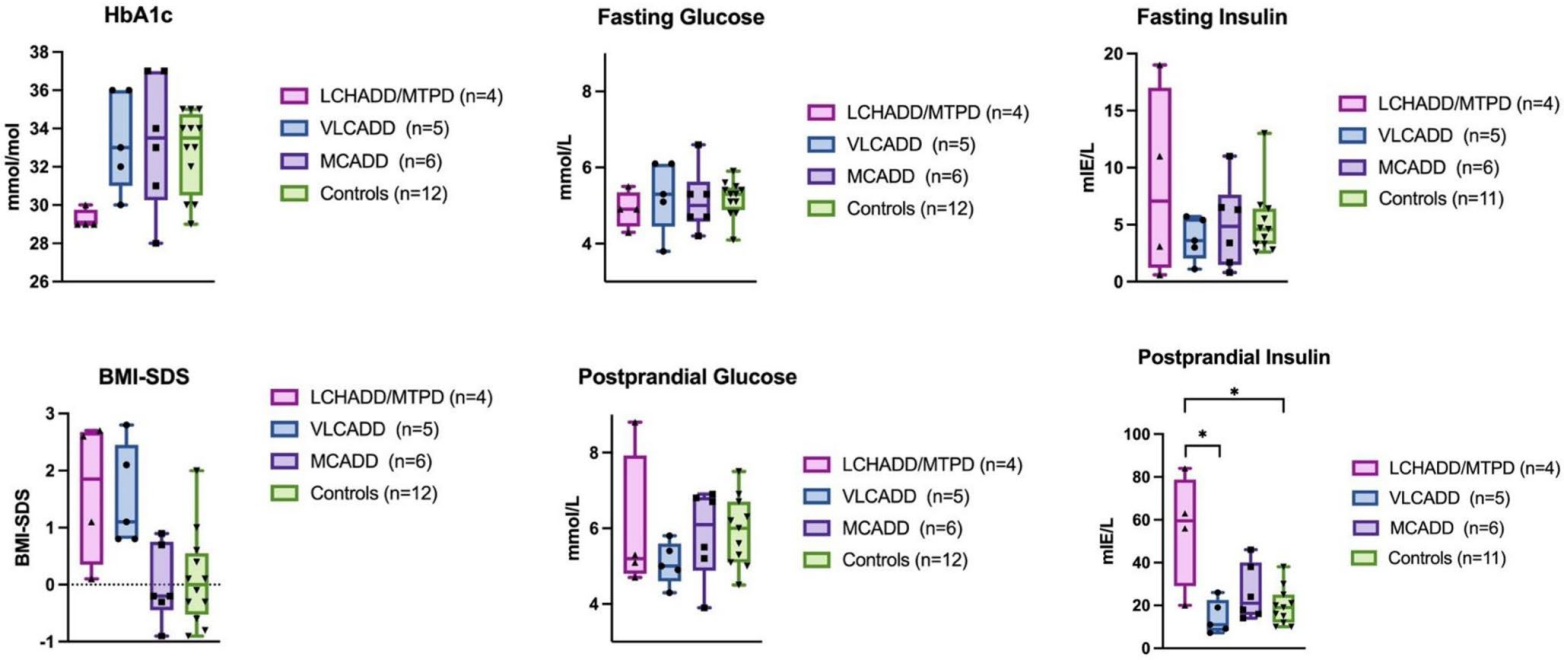
HbA1c, BMI‐SDS, and fasting and postprandial levels of glucose and insulin.

Fasting and postprandial levels of glucose, total amino acids, and branched‐chain amino acids (BCAA) did not differ significantly between groups, and results are summarized in Table 2. One participant in the LCHADD group had a postprandial glucose of 8.8 mmol/L, while all other participants had values below 7.5 mmol/L (Figure 2). This participant with the highest postprandial glucose value also had a higher fasting insulin level (19 mIE/L, normal range 2–25) and postprandial insulin level (84 mIE/L) compared to the other participants that had fasting insulin levels of 0.6–13 mIE/L and postprandial insulin levels of 7.2–63 mIE/L (Figure 2). Ketone levels in the fasted state were 0.1–0.4 mmol/L in patients and 0.1–0.7 mmol/L in controls. All participants had postprandial ketones of 0–0.3 mmol/L (Table 2). The LCHADD group had lower HbA1c than all the other groups; this was significant compared to controls and VLCADD (Figure 2).

**TABLE 2:** Fasting and postprandial analytes.

Analyte	LCHADD		VLCADD		MCADD		Controls	
	Fasting	Postprandial	Fasting	Postprandial	Fasting	Postprandial	Fasting	Postprandial
Glucose (mmol/L)	4.9 (4.3–5.5)	5.2 (4.7–8.8)	5.3 (3.8–6.1)	5.0 (4.3–5.8)	5.0 (4.2–6.6)	6.1 (3.9–6.9)	5.3 (4.1–5.9)	6 (4.5–7.5)
Ketones (mmol/L)	0.2 (0.1–0.2)	0.2 (0.1–0.3)	0.2 (0.1–0.2)	0.2 (0.1–0.2)	0.2 (0.1–0.2)	0.15 (0.1–0.3)	0.2 (0.1–0.7)	0.1 (0–0.3)
NEFA (mmol/L)	0.33 (0.23–0.50)	0.13 (0.07–0.29)	0.31 (0.17–0.48)	0.28 (0.2–0.36)	0.68 (0.28–1.03)	0.25 (0.12–0.46)	0.36 (0.23–0.94)	0.15 (0.06–0.3)
Total AA (μmol/L)	2761 (2490–3098)	3188 (2718–3679)	2858 (2644–2936)	2895 (2379–3003)	2678 (2297–2773)	2867 (2590–3332)	2629 (2235–3645)	2826 (2334–3728)
Total BCAA (μmol/L)	398 (358–423)	495 (394–689)	479 (349–543)	467 (392–559)	335 (233–442)	354 (296–511)	358 (313–592)	406 (348–508)
Total acylcarnitines (μmol/L)	12.3 (10.8–13.2)	12.8 (12.05–13.5)	15.6 (12.8–18.3)	16.0 (13.3–22.5)	14.4 (12.3–30.9)	12.9 (11.9–21.9)	12.8 (10.8–15.5)	13.1 (12.5–14.6)
Disease‐specific acylcarnitines (μmol/L)	C16:OH 0.53 (0.1–0.39) C18:1OH 0.21 (0.08–0.41) C18OH 0.17 (0.1–0.22)	C16:OH 0.18 (0.09–0.23) C18:1OH 0.14 (0.05–0.45) C18OH 0.21 (0.1–0.27)	C14:1 1.55 (0.3–2.8)	C14:1 0.83 (0.3–1.4)	C8 1.65 (0.6–14)	C8 0.97 (0.2–5.3)	—	—
Insulin (mIU/L)	7.1 (0.8–11)	59 (20–84)	4.5 (1.1–6.7)	12 (7–26)	4.9 (0.8–11)	21 (14–46)	4.6 (2.6–13)	19 (10–38)
GH (μg/L)	1.4 (0.05–4.8)	0.3 (0.05–0.4)	0.4 (0.07–11)	2 (0.2–4.3)	1.3 (0.08–10)	0.4 (0.05–0.2)	1.2 (0.06–6)	0.2 (0.05–0.9)
Cortisol (nmol/L)	207 (60–259)	192 (180–237)	186 (120–235)	235 (179–464)	204 (141–252)	234 (126–324)	231 (158–303)	280 (189–525)
Glucagon (pmol/L)	3.92 (1.47–4.32)	4.26 (1.69–7.42)	3.54 (2.4–5.85)	3.84 (2.84–9.07)	3.85 (0.84–0.95)	4.96 (0.40–11.7)	2.85 (0.78–15.2)	4.84 (0.84–10.3)

*Note:* Fasting and postprandial analytes for LCHADD/MTPD (*n* = 4), VLCADD (*n* = 5), MCADD (*n* = 6), and controls (*n* = 11–12).

Fasting NEFA levels for all participants were higher compared to the postprandial levels: 0.38 mmol/L (0.11–1.03) compared to 0.17 mmol/L (0.06–0.46), *p* < 0.001 (Table 2).

Fasting NEFA levels were significantly higher in the MCADD group compared to VLCADD (*p* = 0.030), but the differences did not reach significance compared to the LCHADD and control groups (Table 2).

Fasting acylcarnitine profiles showed an expected pattern with increased levels of disease‐specific species in the LCHADD (C16:1 OH, C16 OH, C18:1 OH, C18 OH), VLCADD (C14:1), and MCADD (C8) groups, which decreased in the postprandial state (Table 2).

### 3.3 Hormone Analyses

Fasting and postprandial levels of insulin, GH, and cortisol showed no significant differences between the groups except for postprandial cortisol, which was lower in LCHADD (192 nmol/L [180–237]) compared to controls (280 nmol/L [189–525]), *p* = 0.041 (Table 2). Fasting insulin levels correlated to age at testing (*r* = 0.42, *p* = 0.028) but not with BMI‐SDS or HbA1c. Postprandial insulin levels tended to be higher in the LCHADD group: 59.5 mIE/L (20–84) compared to VLCADD: 11 mIE/L (7.2–26), *p* = 0.032; MCADD: 21 mIE/L (14–46), *p* = 0.67; and controls: 19 mIE/L (10–38), *p* = 0.020 (Table 2; Figure 2).

Fasting plasma glucagon showed high variability in all groups, but no significant differences were noted between groups (Table 2).

## 4 Discussion

This study shows that children with LCHADD, VLCADD, and MCADD had a normal glycemic response, following a 4‐h fast and after intake of a standardized meal. The changes in most hormones were similar to those observed in the control group.

Glucose homeostasis as measured by HbA1c showed that all patients had values within the normal, nondiabetic range (26–37 mmol/mol) (Figure 2). Interestingly, patients with LCHADD had HbA1c levels in the lower range (29–30 mmol/L) while they tended to have higher fasting and postprandial insulin levels and higher BMI‐SDS. Although we did not find any significant correlation between BMI‐SDS and fasting and postprandial insulin levels, it is possible that the increased insulin resistance seen in these patients was caused by obesity rather than the specific beta‐oxidation disorder. There were no differences in fasting or postprandial glucose levels that correlated to this difference. A conceivable explanation for low HbA1c may be a higher turnover of erythrocytes, possibly due to membrane instability caused by long‐chain 3‐OH fatty acids or alterations of complex lipids, as previously described for mitochondria in murine cardiocytes [27].

BMI‐SDS varied in different groups, and the VLCADD and LCHADD participants had significantly higher BMI‐SDS compared to MCADD and controls (Table 1). Development of obesity is one of the long‐term health risks that have been proposed for patients with beta‐oxidation disorders in general and long‐chain beta‐oxidation disorders in particular [28, 29]. A carbohydrate‐rich diet, shorter fasting intervals compared to the general population, and compensation for the risk of rhabdomyolysis following excessive physical activity could contribute to improper energy balance and weight gain. A study from Poland showed significantly higher BMI‐SDS in a cohort of 39 patients with beta‐oxidation disorder compared to 156 control subjects. However, they did not find any differences between the patients diagnosed by NBS (and thus started on dietary treatment from the neonatal period) compared to those with a clinical diagnosis [30]. In our study cohort, BMI‐SDS for patients with a strict diet overlapped those on a relaxed diet and controls.

The role of FAO for the development of insulin resistance and T2D has been studied in many different contexts. Several mechanisms where decreased FAO leads to increased insulin resistance, such as cellular lipotoxicity and increased ROS activity, have been proposed [17, 31]. Conversely, decreased FAO can lead to decreased insulin resistance due to a switch from fat to glucose utilization [32]. However, patients with LCHADD have been reported to show a normal response to an oral glucose tolerance test [18]. A similar pattern was seen in our study where glucose levels were within the normal range (3.8–6.6 mmol/L) after the 4‐h fast (Table 2). One of the patients with LCHADD had a blood glucose level of 8.8 mmol/L 60 min postprandially in addition to a higher fasting insulin level and a higher postprandial insulin level, suggesting an increased insulin resistance (Figure 2).

Circulating levels of NEFA increase during fasting, and high fasting levels of NEFA have been associated with reduced insulin secretion, T2D, and obesity in children [33, 34]. In this study, no association between high BMI‐SDS and fasting NEFA was seen, and the MCADD group that tended to have higher fasting NEFA levels had lower BMI‐SDS compared to the LCHADD and VLCADD groups.

Disease‐specific acylcarnitines showed large variability in the different disease groups in both the fasted and postprandial states (Table 2). Besides the fasting time and the meal composition, several constitutional factors such as specific beta‐oxidation disorders and residual enzyme activity as well as modifiable factors such as physical activity and carnitine supplementation have been hypothesized to influence the individual acylcarnitine levels [19, 26, 35]. In the VLCADD group, the patients with low residual enzyme activity all had an increased fasting C14:1 level, while the patient with residual activity > 30% had C14:1 within the normal range. The C8 acylcarnitine levels for the patients with MCADD did not correlate to carnitine supplementation in our study (Table 2).

Glucagon, cortisol, and GH are important counter‐regulatory hormones and part of the humoral response to hypoglycemia. The response to decreasing levels of plasma glucose starts with decreased insulin secretion when glucose is within the physiological range (4–7 mmol/L), followed by increased secretion of glucagon and epinephrine and, to a lesser extent, GH and cortisol when glucose falls below 3.9–3.6 mmol/L [36]. While GH and cortisol have diurnal variations and increase as part of the autonomous stress response, secretion of glucagon from the pancreatic alpha‐cells is mainly stimulated by low blood glucose levels and increased FFA [37, 38]. Fasting and postprandial cortisol levels were similar between all groups, while GH showed a high variability (Table 2). Increased fasting levels of glucagon have been shown in children with obesity and impaired glucose tolerance [39]. Stenlid et al. presented data showing lower glucagon levels in children with VLCADD compared to children with MCADD or CUD after a 4‐h fast [16]. In our study, where all participants had standardized meals, fasting glucagon levels were in the same range, and there were no significant differences between participants with VLCADD and the other disease groups or controls (Table 2). Glucagon levels in the 60‐min postprandial sampling were higher compared to fasting levels in all groups and showed high variability. In a study on healthy adults, glucagon levels showed a continued increase at 30 min after a mixed meal and displayed a maximal decrease 2 h postprandially [40]. We found no correlation between an increase or decrease in postprandial glucagon and BMI, diagnosis, age, postprandial glucose, or insulin levels. A possible explanation for these differences could be that the patients were in different phases of the postprandial glucagon trajectory at the time of postprandial sampling.

The limitations of the study are the small number of participants per diagnostic group due to the rarity of the disorders and difficulties in recruiting healthy control participants of a young age. Consequently, there were differences in BMI‐SDS and sex distribution that possibly could affect the outcome. While the caloric intake from breakfast and lunch during the study day was adjusted to age, there were variations in the individual meal nutrient compositions, which were not standardized. Differences in dietary treatment and fasting restrictions, such as LCT restrictions and continuous night feeds, between the groups before the study and in everyday life could not be controlled for. Another potential confounding factor is the variation of genotypes in the study groups, where some study participants in the VLCADD and MCADD groups had attenuated variants. Differences in the time from meal ingestion to blood sampling could also affect postprandial hormone and substrate levels. Even though this did differ slightly between participants due to varied eating habits, we did not observe any systematic differences between groups. This study should be considered as exploratory, and the descriptive findings need to be verified in larger cohorts.

In conclusion, children with LCHADD, VLCADD, and MCADD displayed a normal glycemic response to a 4‐h daytime fast, and the glucose homeostasis was similar to the homeostasis seen in the controls. However, participants with LCHADD who were obese tended to have higher fasting and postprandial insulin levels and lower HbA1c levels. Our results warrant further studies to understand the intricate interplay of carbohydrate and lipid metabolism in beta‐oxidation disorders.

## Author Contributions

Conception and design of the study: David Olsson, Maria Halldin, Charlotte Bieneck Haglind, Svetlana Lajic, and Anna Nordenström. Data acquisition: David Olsson, Anna Nordenström. Interpretation of data: David Olsson, Maria Halldin, Charlotte Bieneck Haglind, Svetlana Lajic, and Anna Nordenström. Drafting the work: David Olsson, Anna Nordenström. Revising the final manuscript critically for intellectual content: David Olsson, Maria Halldin, Charlotte Bieneck Haglind, Svetlana Lajic, Anna Nordenström. Final approval: David Olsson, Maria Halldin, Charlotte Bieneck Haglind, Svetlana Lajic, Anna Nordenström.

## Funding

The study was funded by the Sven Jerring Foundation, Stiftelsen Frimurare Barnhuset i Stockholm, Stiftelsen Wera Ekström, the Karolinska University Hospital, and the Stockholm County Council.

## Ethics Statement

This study was approved by the Ethical Review Board of Uppsala University (registration number 2006:005) and the Swedish Ethical Review Board (PM, 2021‐02305, 2021‐08‐12).

## Consent

Informed consent documents were signed by all legal guardians of the study participants.

## Conflicts of Interest

The authors declare no conflicts of interest.

## Supporting information


**Appendix A1.** Study meal compositions.

## Data Availability

The data that support the findings of this study are available from the corresponding author upon reasonable request.
