# Supplementary material for: Hormonal Responses to a Short Daytime Fast in Children With Beta‐Oxidation Disorders
Source: JIMD Rep. 2026 Aug 23;67(5):e70120. doi: 10.1002/jmd2.70120 (PMC13500227; doi:10.1002/jmd2.70120)
Supplement: Supplementary file 1 [file JMD2-67-e70120-s001.pdf]

Supplementary Appendix A1: Study meal compositions

ID	BREAKFAST Kcal	BF E% CH	BF E% Protein	BF E% LCT Fat	BF E% MCT fat	BF E% Fat	LUNCH Kcal	L E% CH	L E% Protein	L E% LCT Fat	L E% MCT fat	L E% fat
1	110	63	14	10	9	19	330	54	22	12	11	23
2	190	62	17	6	16	22	510	61	21	18	0	18
3	260	69	19	12	0	12	480	65	22	13	0	13
4	220	51	22	4	24	28	440	51	22	4	24	28
5	200	53	18	12	17	29	580	62	18	10	10	20
6	240	59	18	12	11	23	710	63	18	12	7	19
7	190	61	21	10	8	18	410	62	13	14	11	25
8	230	63	23	15	0	15	615	57	31	14	0	14
9	190	58	29	12	0	12	430	52	30	18	0	18
10	180	66	10	23	0	23	446	68	14	18	0	18
11	150	67	8	25	0	25	405	67	14	19	0	19
12	150	58	22	20	0	20	420	58	20	22	0	22
13	150	58	26	20	0	20	420	58	20	22	0	22
14	190	64	23	13	0	13	540	61	21	18	0	18
15	220	62	24	14	0	14	620	61	21	18	0	18
16	180	54	20	26	0	26	540	54	20	26	0	26
17	150	56	18	26	0	26	450	54	20	26	0	26
18	180	54	20	26	0	26	540	54	20	26	0	26
19	185	60	14	22	0	22	510	58	20	22	0	22
20	180	57	31	12	0	12	550	61	15	26	0	26
21	190	64	10	26	0	26	560	61	13	26	0	26
22	260	64	14	23	0	23	660	61	19	20	0	20
23	245	66	13	21	0	21	730	63	18	19	0	19
24	185	63	13	14	0	14	590	61	19	21	0	21
25	165	66	7	27	0	27	520	59	13	27	0	27
26	165	66	7	27	0	27	510	58	15	27	0	27
27	170	63	13	14	0	14	490	58	15	27	0	27
